# Chem(Pro)^2^: the atlas of *chemoproteomic* probes labelling human proteins

**DOI:** 10.1093/nar/gkae943

**Published:** 2024-10-22

**Authors:** Songsen Fu, Zhen Chen, Zhiming Luo, Meiyun Nie, Tingting Fu, Ying Zhou, Qingxia Yang, Feng Zhu, Feng Ni

**Affiliations:** Institute of Drug Discovery Technology, Ningbo University, Ningbo 315211, China; LeadArt Biotechnologies Ltd., Ningbo 315201, China; College of Pharmaceutical Sciences, The Second Affiliated Hospital, Zhejiang University School of Medicine, State Key Laboratory of Advanced Drug Delivery and Release Systems, Zhejiang University, Hangzhou 310058, China; LeadArt Biotechnologies Ltd., Ningbo 315201, China; LeadArt Biotechnologies Ltd., Ningbo 315201, China; College of Pharmaceutical Sciences, The Second Affiliated Hospital, Zhejiang University School of Medicine, State Key Laboratory of Advanced Drug Delivery and Release Systems, Zhejiang University, Hangzhou 310058, China; College of Pharmaceutical Sciences, The Second Affiliated Hospital, Zhejiang University School of Medicine, State Key Laboratory of Advanced Drug Delivery and Release Systems, Zhejiang University, Hangzhou 310058, China; Zhejiang Provincial Key Laboratory of Precision Diagnosis and Therapy for Major Gynecological Diseases, Women's Hospital, Zhejiang University School of Medicine, Hangzhou 310058, China; College of Pharmaceutical Sciences, The Second Affiliated Hospital, Zhejiang University School of Medicine, State Key Laboratory of Advanced Drug Delivery and Release Systems, Zhejiang University, Hangzhou 310058, China; Innovation Institute for Artificial Intelligence in Medicine of Zhejiang University, Alibaba-Zhejiang University Joint Research Center of Future Digital Healthcare, Hangzhou 330110, China; Institute of Drug Discovery Technology, Ningbo University, Ningbo 315211, China; LeadArt Biotechnologies Ltd., Ningbo 315201, China

## Abstract

*Chemoproteomic* probes (CPPs) have been widely considered as powerful molecular biological tools that enable the highly efficient discovery of both binding proteins and modes of action for the studied compounds. They have been successfully used to validate targets and identify binders. The design of CPP has been considered extremely challenging, which asks for the generalization using a large number of probe data. However, none of the existing databases gives such valuable data of CPPs. Herein, a database entitled ‘Chem(Pro)^2^’ was therefore developed to systematically describe the atlas of diverse types of CPPs labelling human protein in living cell/lysate. With the booming application of *chemoproteomic* technique and *artificial intelligence* in current chemical biology study, Chem(Pro)^2^ was expected to facilitate the AI-based learning of interacting pattern among molecules for discovering innovative targets and new drugs. Till now, Chem(Pro)^2^ has been open to all users without any login requirement at: https://idrblab.org/chemprosquare/

## Graphical Abstract

**Graphical Abstract:**
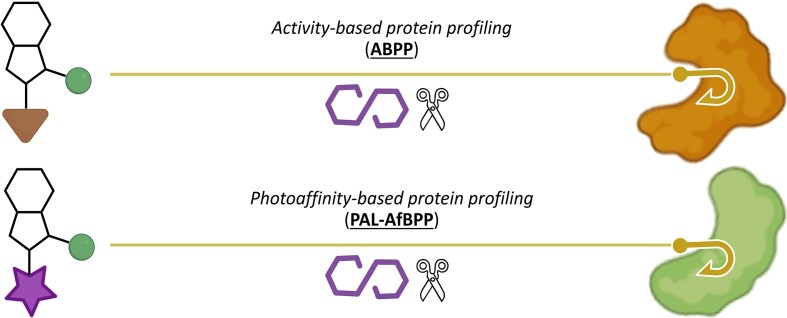

## Introduction

Chemoproteomic probes (CPPs) have been widely considered as a powerful molecular biological tool that enables the highly efficient discovery of both binding proteins and modes of action for studied compounds (1–3). They have been successfully applied in various contexts, including identifying/validating drug targets by mapping compound-protein interactions (4–8), screening lead compounds in early drug discovery (9–12) and optimizing the efficacy/specificity of the identified leads (13–16). It is known that the design of CPP remains extremely challenging, which asks for the generalization based on a large number of probe data (17). All the studies highlight the demands for the valuable information of CPP, and it is therefore critical to collect the *big data* of CPP for promoting the AI-based learning of interacting patterns among molecules for discovering innovative targets and drugs (18–27).

So far, a variety of databases have been developed to provide the interactions between compound and protein, including BindingDB (28), ChEMBL (29), CovPDB (30), GtoPdb (31), KLIFS (32), Chemical Probes Portal (33) and CovalentInDB (34). Only one database named CysDB (35) has been available for describing CPP data, which focuses on offering three CPPs covalently binding to the *cysteine* sites of 11 621 human proteins detected using the *chemoproteomic* technique, and 363 competitive compounds (namely, *competitors*) corresponding to the CPPs were accumulated using experimentally-validated data. Most of the existing databases have attracted broad interest from worldwide audiences and won substantial reputation from the relevant research community, which further highlights the great importance of the accumulation of these valuable CPP data.

However, it is known that the probes are designed to covalently bind to any amino acids (not just cysteine) of the human proteins, and the diversity of the binding amino acid is of great importance for the identification of the target/drug (36–42). Besides the covalent ones, photoaffinity probes are known to be appealing and promising to noncovalent drugs for the enrichment of targets from the whole cellular proteome (43,44), and it is critical to have the binding data of probes validated in living cell to represent the complexity of the biological system (45–48). To the best of our knowledge, none of the existing databases provided such valuable information as described above. Therefore, it is urgently needed to have a knowledge base that systematically describes the precious data of diverse types of *chemoproteomic probes* (CPPs) labelling the human proteins in living cells.

Herein, a database named Chem(Pro)^2^, which systematically describes the atlas of *chemoproteomic probes* (CPPs) labelling human proteins in living cells was therefore developed. First, CPPs were collected based on comprehensive literature reviews in PubMed, which led to a total of 603 CPPs (133 covalent and 470 photoaffinity), 1016 *competitors* of CPPs and 14 250 binding proteins in the human genome (including 4649 enzymes, 1357 channel/transporters, 225 GPCRs and so on). Second, all interactions between CPPs and their labelling human proteins were experimentally discovered based on a total of 118 living cell types (such as HEK293T, HCT-116, PaTu-8988t, MDA-MB-231, HeLa) from 22 healthy/disease organs (including kidney, colon, uterus, breast, pancreas, and liver). Third, the information on the probe's binding sites in human protein was comprehensively collected from the literatures and explicitly provided in Chem(Pro)^2^, which resulted in a total of 135 486 binding sites in 13 938 human proteins labelled by 160 CPPs. Finally, the binding ratios between 524 CPPs and 863 *competitors* (which challenge the labelling of CPPs on proteins) were collected, which brought about 2 118 636 records of binding ratio experimentally validated based on 106 living cell types (including K562, Hep-G2, Ramos, SH-SY5Y and KYSE410).

All in all, Chem(Pro)^2^ is unique in systematically providing diverse types of *chemoproteomic probes* (both the covalent and photoaffinity ones) that experimentally label human proteins in living cells. Its provided data were fully cross-linked to many established molecular biological knowledge bases, such as UniProt (49), PubChem (50), TTD (51), DrugMAP (52) and ChEMBL (29). With the booming applications of *chemoproteomic techniques* in chemical biology research, our database is therefore expected to attract broad interests from the relevant research community. The Chem(Pro)^2^ is now freely accessible by all users at: https://idrblab.org/chemprosquare/.

## Factual content and data retrieval

### Data collection for *chemoproteomic* probes (CPPs), competitors and targets

The data for Chem(Pro)^2^ were collected through the following procedures. Particularly, a literature review was conducted using keywords and combinations such as ‘chemoproteomics + probes’, ‘chemoproteomics + targets’, ‘activity-based protein profiling’, ‘affinity-based protein profiling’, ‘photoaffinity labelling’ and ‘click chemistry’ in PubMed and PRIDE (53). Entries detailing reported probes along with their corresponding target were documented. As a result, Chem(Pro)^2^ established a comprehensive *chemoproteomic atlas* that integrated a diverse series of CPPs with their labelling targets and *competitors*. Experimentally, each studied CPP can label multiple targets and be competed by several competitors, which established the relationship among the three main components in Chem(Pro)² database: CPP, *competitor*, and target.

**Figure 1.**
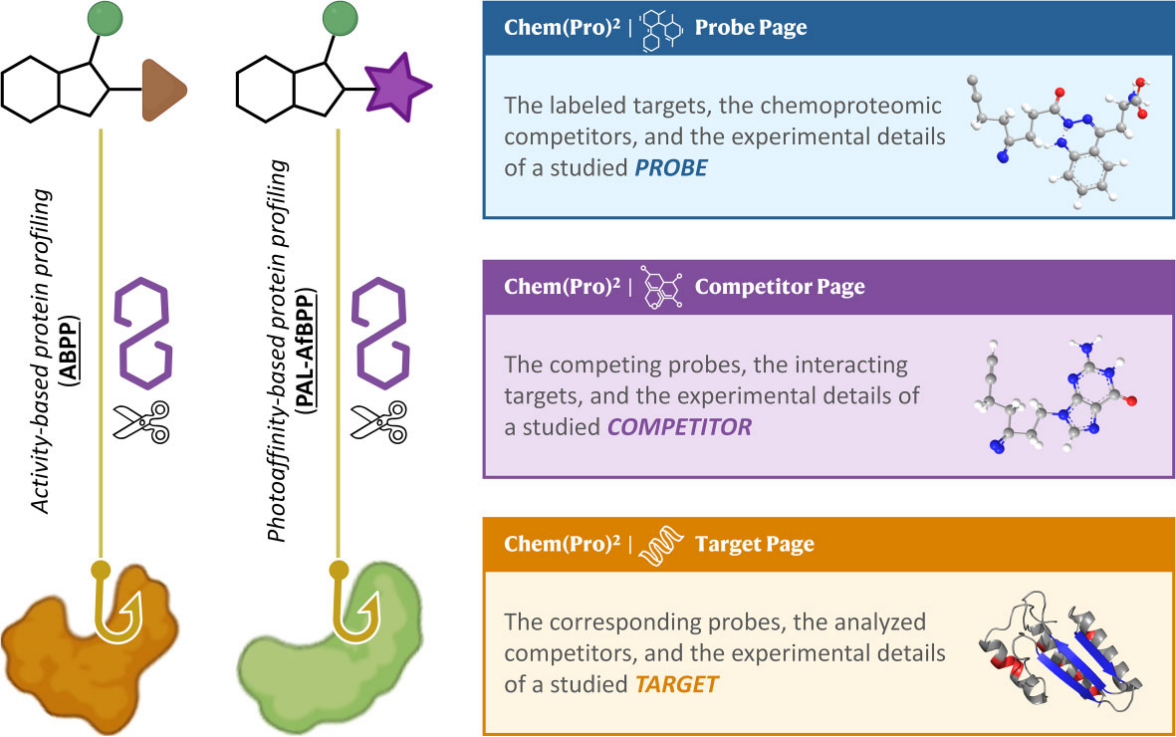
The relationships among the three key components in our Chem(Pro)² database: CPPs, *competitors* and probe labelling targets. One CPP can label many targets, and specific competitor can compete with the CPP when binding to targets. As described on the left side, there were two types of probe-associated techniques: *activity-based protein profiling* (ABPP) and *photoaffinity-based protein profiling* (PAL-AfBPP). As demonstrated on the right side, the primary content in each of the Chem(Pro)² page (the pages for *Probe*/*Competitor*/*Target*) was also provided.

As depicted on the left side of Figure 1, there were two typical types of probe-protein profiling technique: *activity-based protein profiling* (ABPP (54)) and *photoaffinity-based protein profiling* (PAL-AfBPP (55)). The probes in Chem(Pro)^2^ were specifically designed for labeling and enriching the target protein profiles in proteomics analyses and the key differences between probes in Chem(Pro)^2^ and the Chemical Probes Portal was shown in Supplementary Table S1. Moreover, the main contents in the Chem(Pro)² pages describing those three components were briefly provided (the right side of Figure 1), which included the labeled targets, *chemoproteomic competitors*, and experimental detail of the studied probe, the competing probes, interacting targets, and experimental detail of a studied competitor, and the corresponding probes, analyzed competitors, and experimental detail of the studied target. In Chem(Pro)^2^, readers could readily retrieve the information of the three main components and their one-to-many relationship, and 603 CPPs (133 covalent and 470 photoaffinity), 1016 *competitors* and 14 250 protein targets (such as 4649 enzymes and 1357 channel/transporters) were finally included into Chem(Pro)².

**Figure 2.**
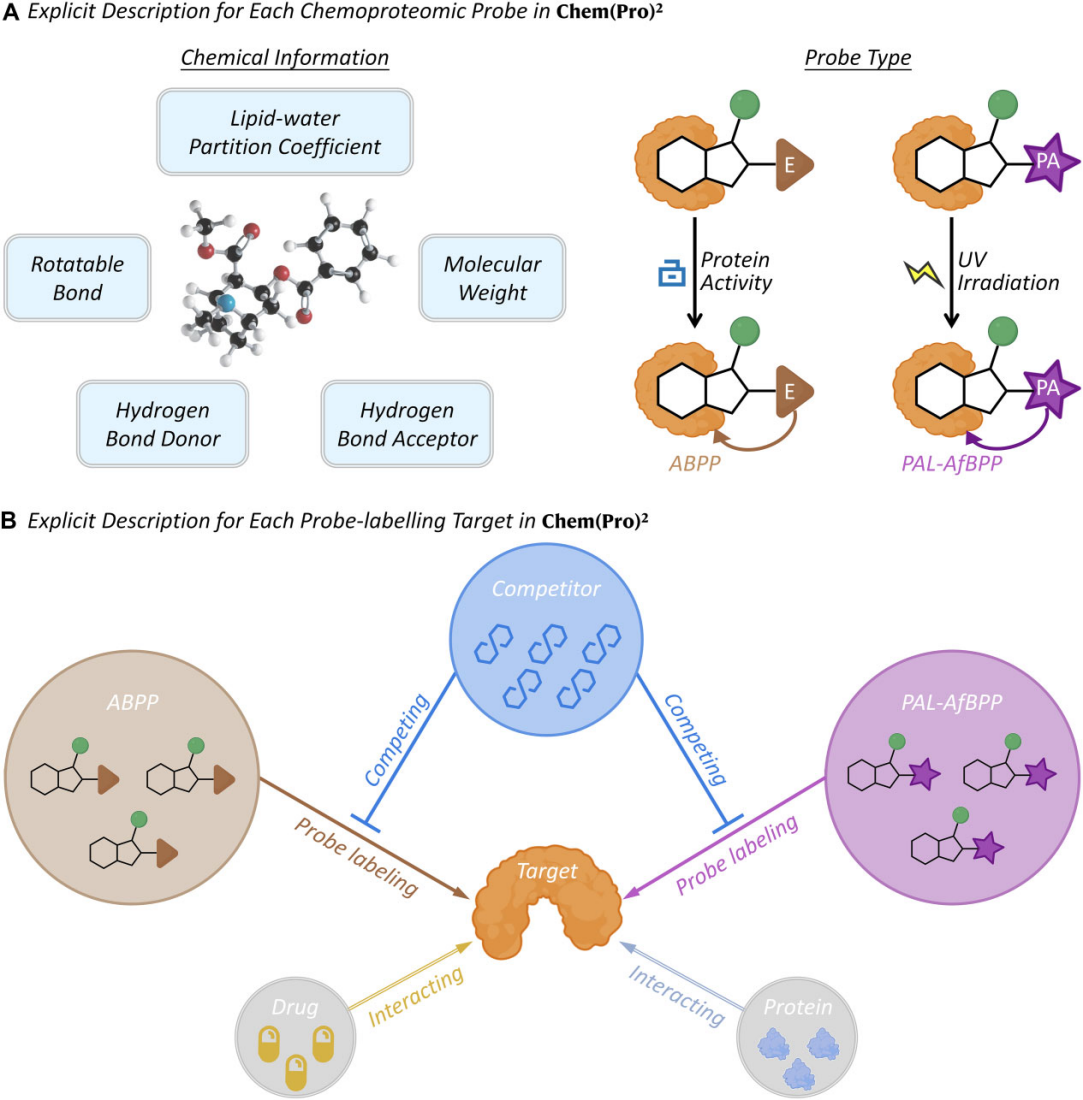
Detailed information offered in Chem(Pro)² for describing the *chemoproteomic* probes (CPPs) and their labelling targets. (**A**) explicit descriptions for each CPP in Chem(Pro)² database, which included various chemical data (such as lipid-water partition coefficient, molecular weight, hydrogen bond donor, hydrogen bond acceptor, and rotatable bond) and diverse probe techniques (ABPP and PAL-AfBPP). (**B**) explicit descriptions for every probe-labelling target in Chem(Pro)², which described the competing mechanism of *competitors* underlying each probe technique, and a variety of drugs and proteins that interacted with the studied probe-labelling target.

Furthermore, a variety of detailed information was incorporated into our Chem(Pro)² to describe the *chemoproteomic* probes and their labelling target. As depicted in Figure 2A, *chemoproteomic* probes in Chem(Pro)² were systematically described by various chemical data (such as lipid-water partition coefficients, molecular weight, rotatable bond, hydrogen bond donor and hydrogen bond acceptor) and probe techniques (ABPP and PAL-AfBPP). Meanwhile, the probe-labelling targets were also explicitly introduced and shown in Figure 2B. Chem(Pro)² introduced all interactions and labelling processes involving a studied probe, and the competing mechanisms of *competitors* underlying different probe techniques were also shown. Moreover, a large number of drugs (2033 approved, 2379 clinical trial, 3588 preclinical/patented and 9631 investigative ones) and 10 533 proteins interacting with the probe-labelling targets were also collected.

### Activity-based protein profiling (ABPP) probes labelling the protein targets

ABPP is a well-used *chemoproteomic* technique initially developed to monitor enzyme activities in complex proteomes (56–63). Various types of enzymes have been targeted using ABPP probes, which include proteases, kinases, phosphatases, glycosidases, oxidoreductases, and so on (64–67). To overcome the limitation of traditional protein profiling techniques, broad-spectrum ABPP probes with highly reactive warheads have been designed to label the intrinsically reactive residues across various protein families (68). For example, the iodoacetamide-derived probe ‘*IA-alkyne*’ labelled over 1000 functional cysteines in human cells (69), which included a list of hyper-reactive cysteines associated with enzyme's functional sites, highlighting the versatility of broad-spectrum probes. In addition to the large number of probes targeting cysteine (70), other amino acid residues, such as lysine, tyrosine, aspartic acid, glutamic acid, histidine, tryptophan and methionine, have also been frequently adopted in current *chemoproteomic* studies (71–74). The key features of the probes included (a) a chemically reactive warhead that covalently binds to a specific residue/cofactor on the labelled target, (b) a reporter tag for detecting/purifying the labelled targets and/or (c) a binding group that fine-tunes target-labelling specificity and minimizing undesirable interactions between the warhead and the tag.

**Figure 3.**
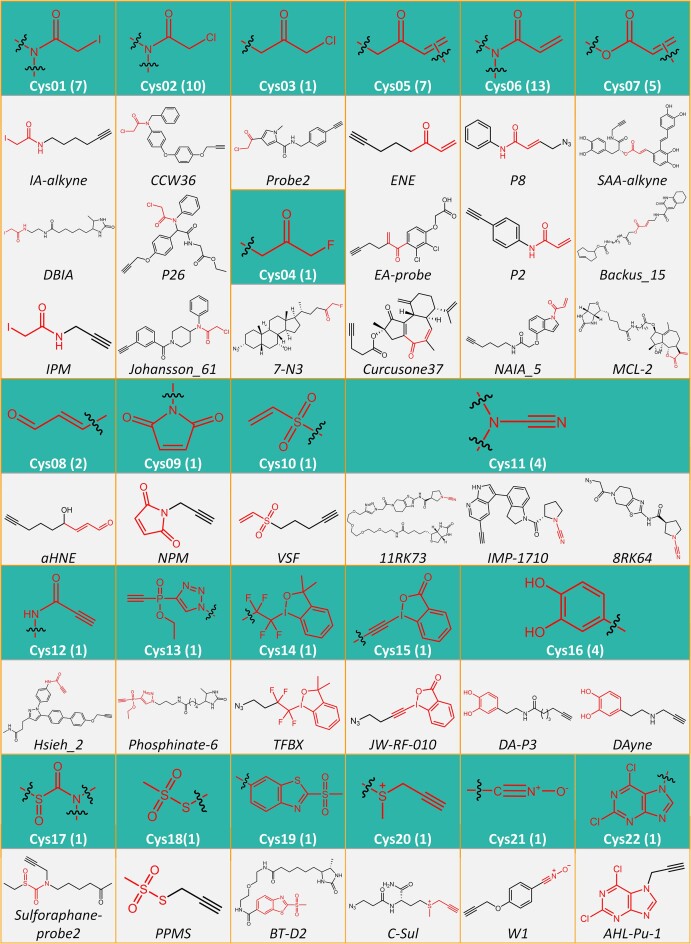
The atlas of the *activity-based protein profiling* (ABPP) probe labelling cysteine. There were 22 types of ABPP probes under this category (from Cys01 to Cys22, highlighted in green background, the number in bracket denoted the total amount of ABPP probes within certain probe type). Under each probe type, both structure and name of the representative probes were provided, and the corresponding probe warhead was highlighted in red color. A square frame in orange was used to highlight the specific information for each cysteine-labelling probe type.

In Chem(Pro)^2^, a comprehensive collection of ABPP probes along with their labelled targets was provided. As one of the most popular labelling residues, cysteine was reported to be the only one covalently labelled by marketed drugs, which highlighted its dominant role in *chemoproteomic* research. In this study, the atlas of all the cysteine-labelled ABPP probes collected to Chem(Pro)^2^ was systematically described in Figure 3, which resulted in 22 types of ABPP probe (from Cys01 to Cys22, highlighted in green background, the numbers in brackets indicated the total number of collected probes in the corresponding probe type). In each probe type, both the structure and name of the representative ABPP probes were illustrated, with the corresponding warhead highlighted in red color. A number of square frames in orange were used to highlight certain data for each cysteine-labelled probe type. Particularly, the commonly-used warheads (colored in red) included electron-deficient haloalkane or electron-deficient alkene, leading to the development of diverse ABPP probes, such as *ENE* (17), *AZ-9* (36), *IA-alkyne* (69), *IPM* (75), *DBIA* (76,77) and *NAIA_5* (78). Other warheads incorporated many chemotypes: (a) electron-deficient alkyne probes (such as *Hsieh_2* (79), and *phosphinate-6* (80)), (b) benziodoxole probes (such as *TFBX* (81) and *JW-RF-010* (82)), (c) catechol probes (such as *DA-P3* (83), and *DAyne* (84)), (d) sulfonium probes (such as *C-Sul* (85)), (e) nitrile oxide probes (such as *W1* (86)) and so on.

**Figure 4.**
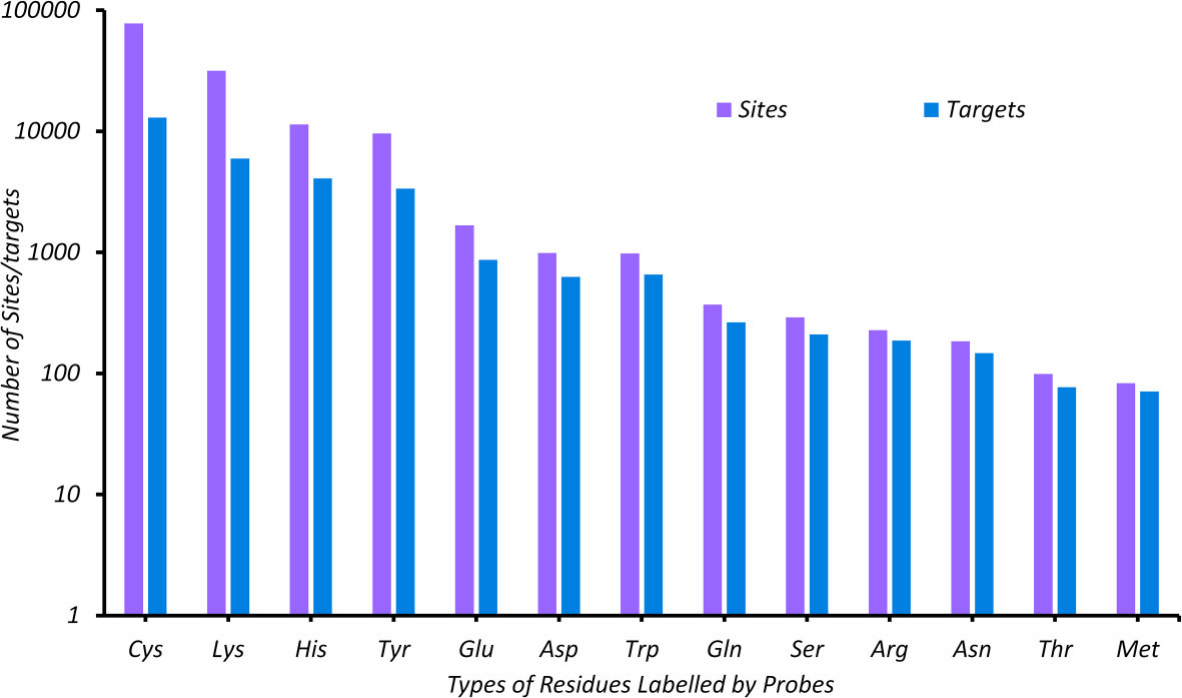
Different numbers of probe-labelled sites and targets according to the various types of residues labelled by all probes described by Chem(Pro)². Among all existing amino acids, a total of thirteen amino acids were identified in this study to be labelled by at least one *chemoproteomic* probe, which made Chem(Pro)² covering the most comprehensive types of probe-labelled amino acids among available databases. Particularly, the *cysteine* was the most popular one labelled by probe, while the *methionine* was the one with the least numbers of sites and targets.

Additionally, many CPPs in Chem(Pro)^2^ database were discovered to label the post-translational modification of cysteine. As provided in Supplementary Figure S1, these CPPs belonged to four different classes: 4-hydroxy-2-nonenal-modified cysteine (*Cys-HNE*), acrolein-modified cysteine (*Cys-Acrolein*), sulfenylation of cysteine (*Cys-SOH*) and sulfination of cysteine (*Cys-SO2H*). Taking the type of *Cys-SOH* as an example, there were three types: (a) Wittig reagent probes (such as *WYneX* (87)), (b) benzothiazine probes (such as *BTD* (88)), (c) dimedone probes (such as *DYn-2* (89)). Except for cysteine and its post-translational modifications, the ABPP probes for other amino acids were also collected to Chem(Pro)^2^. As shown in Supplementary Figure S2, the atlas of *activity-based protein profiling* (ABPP) probes labelling the resides beyond cysteine and its post-translational modification was systematically described, with detailed information was explicitly discussed in the corresponding figure legend. All in all, Chem(Pro)^2^ provided 135 486 ABPP probe-labelled sites relevant to 13 amino acids in 13 938 human proteins (as shown in Figure 4), which made it the most comprehensive repository of probe-labelled amino acids among existing knowledge bases.

### Photoaffinity-based protein profiling (PAL-AfBPP) probes labelling targets

Most FDA-approved drugs bind non-covalently to protein targets. Tools for evaluating reversible interactions between small molecules and proteins are invaluable in this context. To address this need, the *affinity-based protein profiling* (AfBPP) strategy incorporated with *photoaffinity labelling* (PAL) (90) was developed. PAL-AfBPP probe (commonly known as the *photoaffinity-based protein profiling* probe) has become a versatile tool for mapping the reversible interactions between small molecules and proteins in living cells, aiding in the identification of protein targets for various molecules (such as lead compounds and natural product (91–93)), which significantly expanded the spectrum of ‘ligandable’ proteins (94–97). A typical PAL-AfBPP probe consists of three essential components: a scaffold that facilitates the probe's approach to the targeted proteins, a photoreactive group that forms a covalent bond with the target upon exposure to radiation, and a reporter tag for subsequent detection or enrichment. Typical photoreactive groups included aryl azides, diazo ketones, diazirines, benzophenones and aryl tetrazoles, with diazirines being favored due to high reactivity and compact size. While the type and attached sites of the photoreactive group impacted labelling profile of scaffold (98,99), the overall landscape of protein labelling was predominantly determined by the diversity of scaffold structure. Till now, a large number of PAL-AfBPP probes and their labelled proteins (95,96) have been generated within the research community, making it an opportune time to construct a comprehensive database, which would give substantial insight into the design of innovative *chemoproteomic* probes and the discovery of new therapeutics.

**Figure 5.**
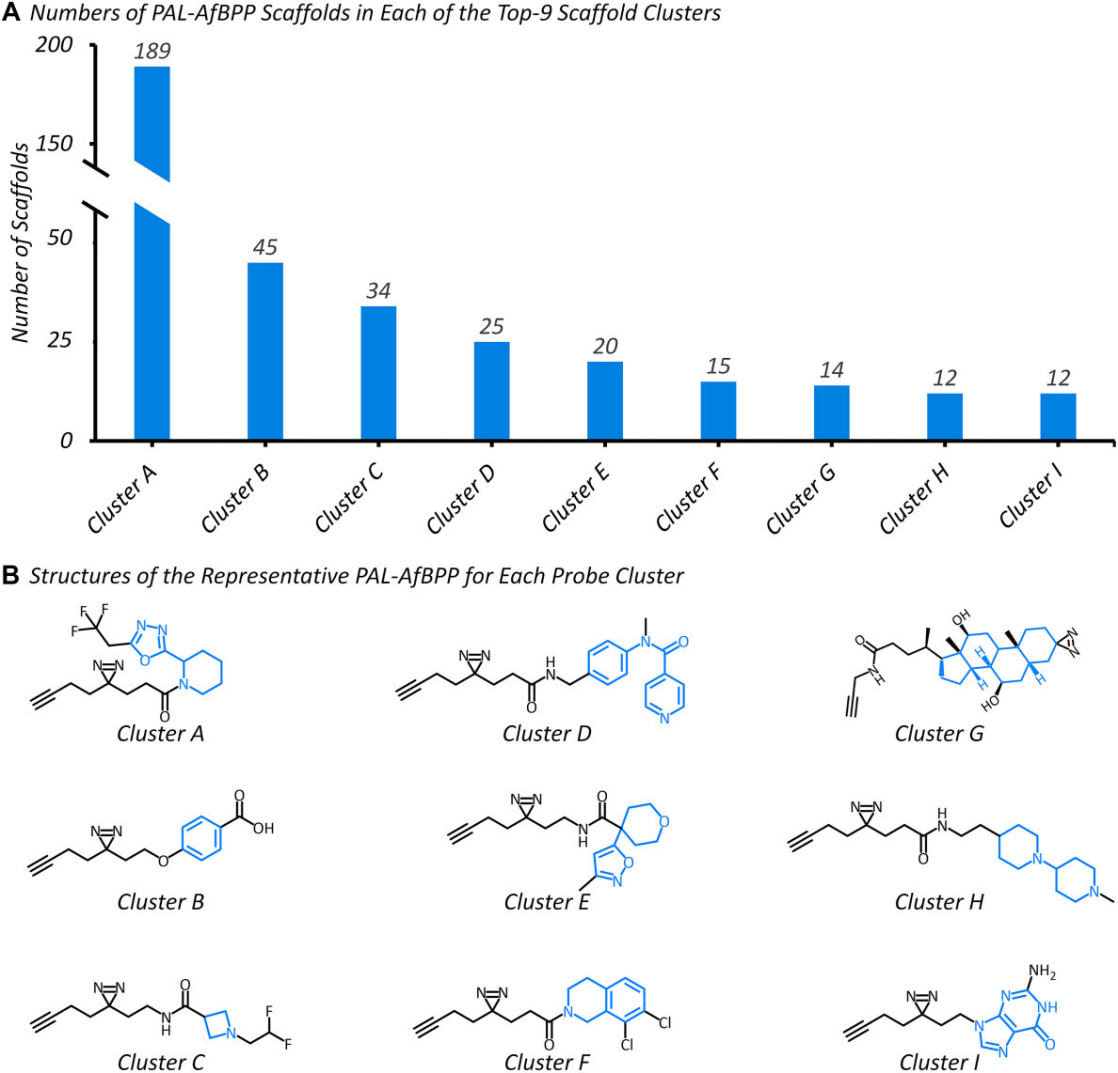
The number and structure of *photoaffinity-based protein profiling* (PAL-AfBPP) probe scaffold in different scaffold clusters. (**A**) the number of PAL-AfBPP scaffolds in each of the top-9 scaffold cluster. (**B**) the structure of the representative PAL-AfBPP probe for each probe cluster, and the corresponding scaffold was highlighted in blue color.

The latest Chem(Pro)^2^ contained 488 PAL-AfBPP probes labelling 4 864 human proteins. Given the importance of scaffold diversity, the structure variety of all accumulated scaffolds was further investigated. First, RDKit package (100) was utilized to generate *Murcko scaffolds* and compute *Morgan fingerprints* for all probes, and the similarity among probes was calculated using *Tanimoto* coefficients (101). Second, based on these similarity scores, hierarchical clustering of all probes was performed using a linkage strategy to calculate the distances among clusters with the threshold set to 0.85 (102). Third, within each cluster, the representative scaffolds were discovered by calculating the average fingerprint and identifying the scaffold closest to this average. Finally, a total of 35 clusters were generated, and the numbers of PAL-AfBPP scaffolds in each of the top-9 scaffold clusters were provided in Figure 5A. Moreover, the structures of the representative PAL-AfBPP probe scaffolds within those top-9 clusters were described in Figure 5B, which demonstrated considerable structure diversity among all the collected probes.

### Target diversity in functional and clinical relevance

**Figure 6.**
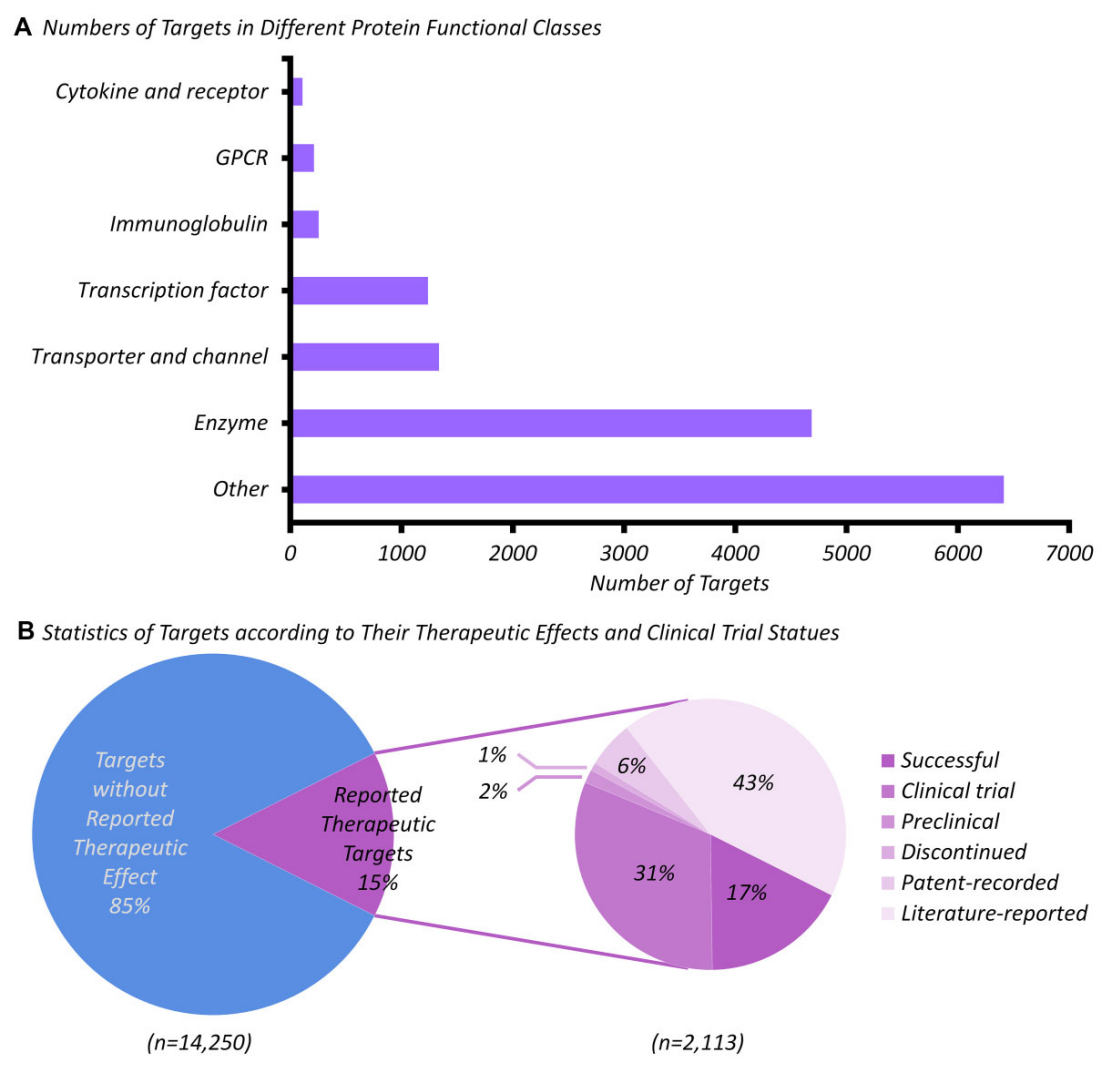
A systematic classification of probe-labelled targets. (**A**) the numbers of targets among a variety of protein functional classes. (**B**) the percentages of targets according to their therapeutic effects and clinical trial status. Particularly, about 15% of the probe-labelled targets had already been previously reported as ‘therapeutic target’, which could be further classified into successful, clinical trial, preclinical, discontinued, patent-recorded and literature-reported ones.

Chem(Pro)^2^ collected a total of 14 250 probe-labelling targets. To have a deeper understanding of these targets, both functional and clinical relevance diversities of the labelled protein targets were explicitly depicted in Figure 6A and B, respectively. Particularly, the targets fell in different protein functional classes, such as enzymes, transporters/channels, transcription factors, immunoglobulins, GPCRs, and cytokines/cytokine receptors (as demonstrated in Figure 6A). Furthermore, all probe-labelling targets were mapped to a well-known database TTD (103), which described the statistics of those targets based on their therapeutic effects and clinical trial status (as illustrated in Figure 6B). Specifically, 15% of those targets were reported therapeutic ones, among which 17% and 31% were the ones of approved (successful) and clinical trial drugs, respectively. For the targets of clinical importance, those established CPPs could play critical role in evaluating target occupancy for drugs associated with preclinical or clinical targets, as well as facilitating ligand screening for literature-reported target. For those targets yet to enter the clinical trials, the CPPs in Chem(Pro)^2^ could also be proactively employed for promoting the studies of ligand screening, thereby capturing the opportunities for lead/drug discovery.

### Functional search engines for retrieving the probe, competitor, and target

**Figure 7.**
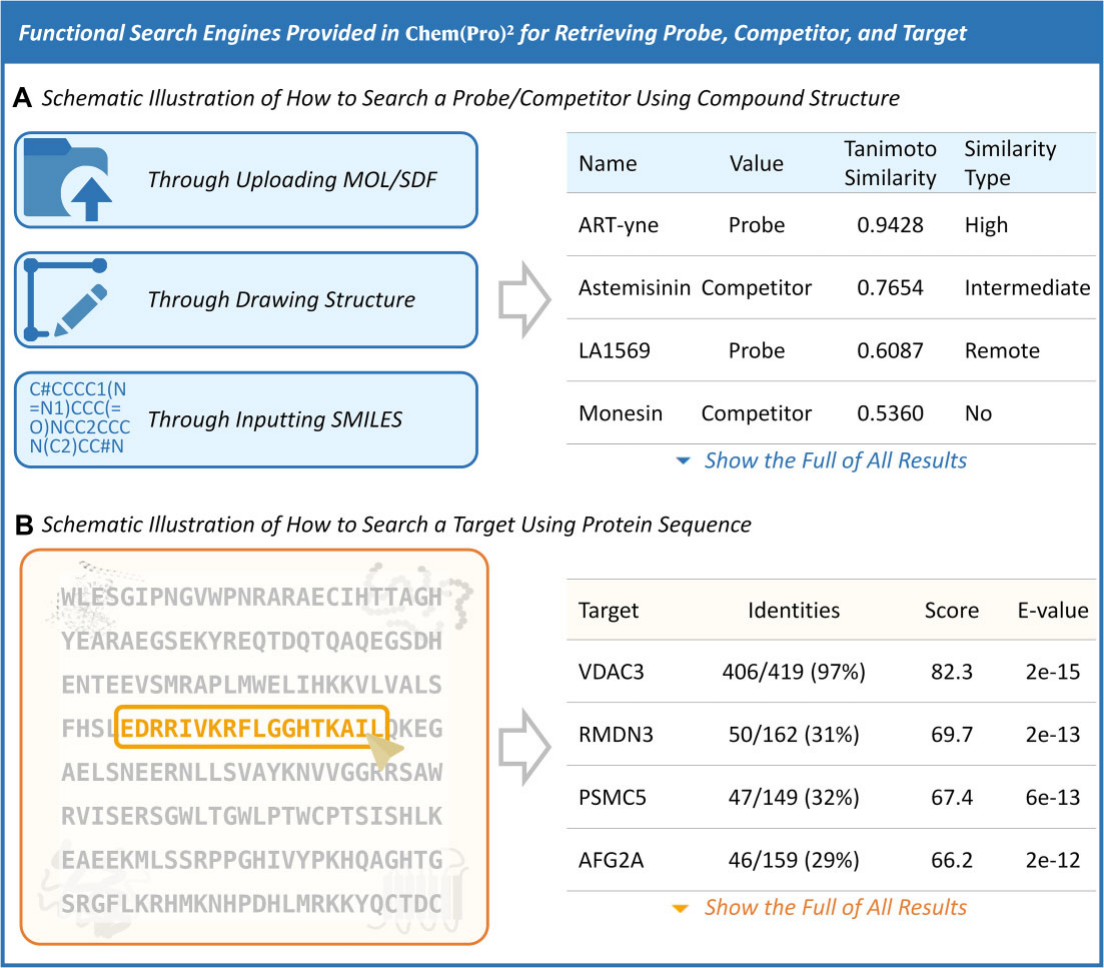
The functional search engines provided in Chem(Pro)² for retrieving probe, competitor, and target. (**A**) a schematic illustration of how to search a probe/competitor based on user-defined compound structures. Chem(Pro)² engine could output the compound similarity by matching the user-defined one to all the probe/competitor structures in our database, and a variety of similarity degrees were calculated (High Similarity, Intermediate Similarity, and Remote Similarity). (**B**) a schematic illustration of how to search probe-labelled target using user-defined protein sequence. Chem(Pro)² engine could calculate the sequence similarity by aligning the user-defined sequence against all target sequences collected to our database, and similarity levels were measured using the BLAST *E-values* and *Identities* as the quantitative similarity scores.

A variety of search engines were constructed in this study and provided online in our Chem(Pro)² for retrieving the information of probe, competitor and target. Chem(Pro)^2^ has developed a keyword-based search engine that allows users to search for drugs, competitors, and targets across the entire database. For example, in the ‘Search for Probe’ interface, users can directly search for probes by name (e.g. IA alkyne, AZ-9, A-DA), or retrieve a list of probes associated with specific targets (e.g. SLC25A20) or competitors (e.g. JZ128). What's more, Figure 7A schematically illustrates the way to search for a probe/competitor based on the user-defined compound structures. Particularly, the provided engine could output the compound similarities by matching the user-defined one to all probe/competitor structures in Chem(Pro)² database, and a variety of similarity degrees could then be calculated, and classified into different levels of similarity (High Similarity, Intermediate Similarity and Remote Similarity). Meanwhile, Figure 7B further provided the way to search probe-labelled targets based on the user-defined protein sequence. Chem(Pro)² engine could calculate sequence similarities by aligning the user-defined sequence against all target sequences collected to the database, and similarity levels were measured using the BLAST *E-values* and *Identities* as the quantitative similarity scores (104). All in all, by integrating such sophisticated measurements, our database was expected to streamline the search procedure by offering powerful and user-friendly interfaces that aimed at supporting efficient data retrieval and scientific data analysis.

## Supplementary Material

gkae943_Supplemental_File

## Data Availability

Chem(Pro)^2^ is open to all users without login requirement at: https://idrblab.org/chemprosquare/.
