# Supplementary material for: Chem(Pro)2: the atlas of chemoproteomic probes labelling human proteins
Source: Nucleic Acids Res. 2024 Oct 22;53(D1):D1651–62. doi: 10.1093/nar/gkae943 (PMC11701659; doi:10.1093/nar/gkae943)
Supplement: gkae943_Supplemental_File [file gkae943_supplemental_file.pdf]

***Supplementary Information for*:**

Chem(Pro)^2: the atlas of *chemoproteomic* probes labelling human proteins

Songsen Fu[1,2,†], Zhen Chen[3,†], Zhiming Luo[2], Meiyun Nie[2], Tingting Fu[3], Ying Zhou[3], Qingxia Yang[4,*], Feng Zhu[3,5,*], Feng Ni[1,2,*]

[1] Institute of Drug Discovery Technology, Ningbo University, Ningbo 315211, China.

[2] LeadArt Biotechnologies Ltd., Ningbo, 315201, China.

[3] College of Pharmaceutical Sciences, The Second Affiliated Hospital, Zhejiang University School of Medicine, State Key Laboratory of Advanced Drug Delivery and Release Systems, Zhejiang University, Hangzhou 310058, China.

[4] Zhejiang Provincial Key Laboratory of Precision Diagnosis and Therapy for Major Gynecological Diseases, Women's Hospital, Zhejiang University School of Medicine, Hangzhou, 310058, China.

[5] Innovation Institute for Artificial Intelligence in Medicine of Zhejiang University, Alibaba-Zhejiang University Joint Research Center of Future Digital Healthcare, Hangzhou 330110, China.

* To whom correspondence should be addressed. Prof. Feng Ni (nifeng@nbu.edu.cn); Prof. Feng Zhu (zhufeng@zju.edu.cn); Prof. Qingxia Yang (yangqx@zju.edu.cn)

† These authors contributed equally to this work as co-first authors.

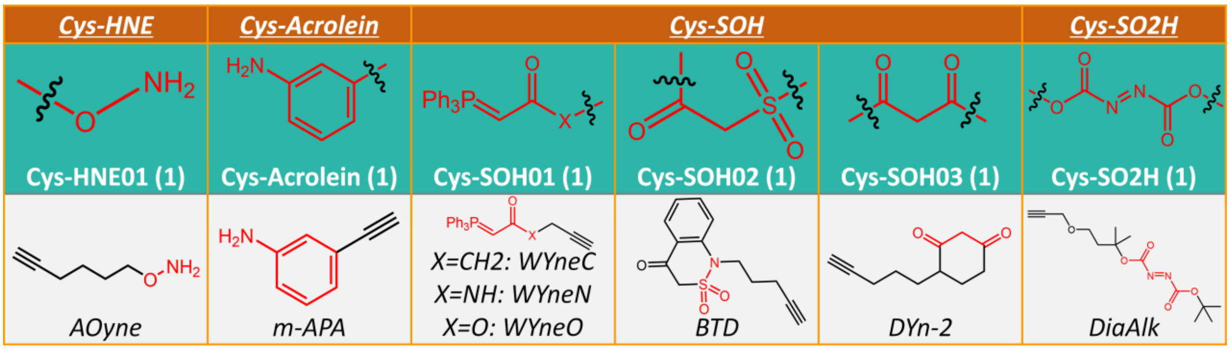

Figure S1. Atlas of *activity-based protein profiling* (ABPP) probe labelling the post-translational modification of cysteine. There were 6 types of ABPP probe (highlighted in *GREEN* background, the number in bracket indicated the total amount of ABPP probes within certain probe type) from four classes (highlighted in *BROWN* background and *Italic* & *Underline* font). Such classes were 4-hydroxy-2-nonenal-modified cysteine (*Cys-HNE*), acrolein-modified cysteine (*Cys-Acrolein*), sulfenylation of cysteine (*Cys-SOH*) and sulfination of cysteine (*Cys-SO2H*). Taking the type of *Cys-SOH* as an example, there were three typical types (***a***) wittig reagent probes (such as *WYneX*), (***b***) benzothiazine probes (such as *BTD*), and (***c***) dimedone probes (such as *DYn-2*). For each of those types, both structure and name of representative probes were shown, and the corresponding probe warhead was highlighted in *RED* color. A square frame in *ORANGE* was used to highlight the specific information for each ABPP probe type.

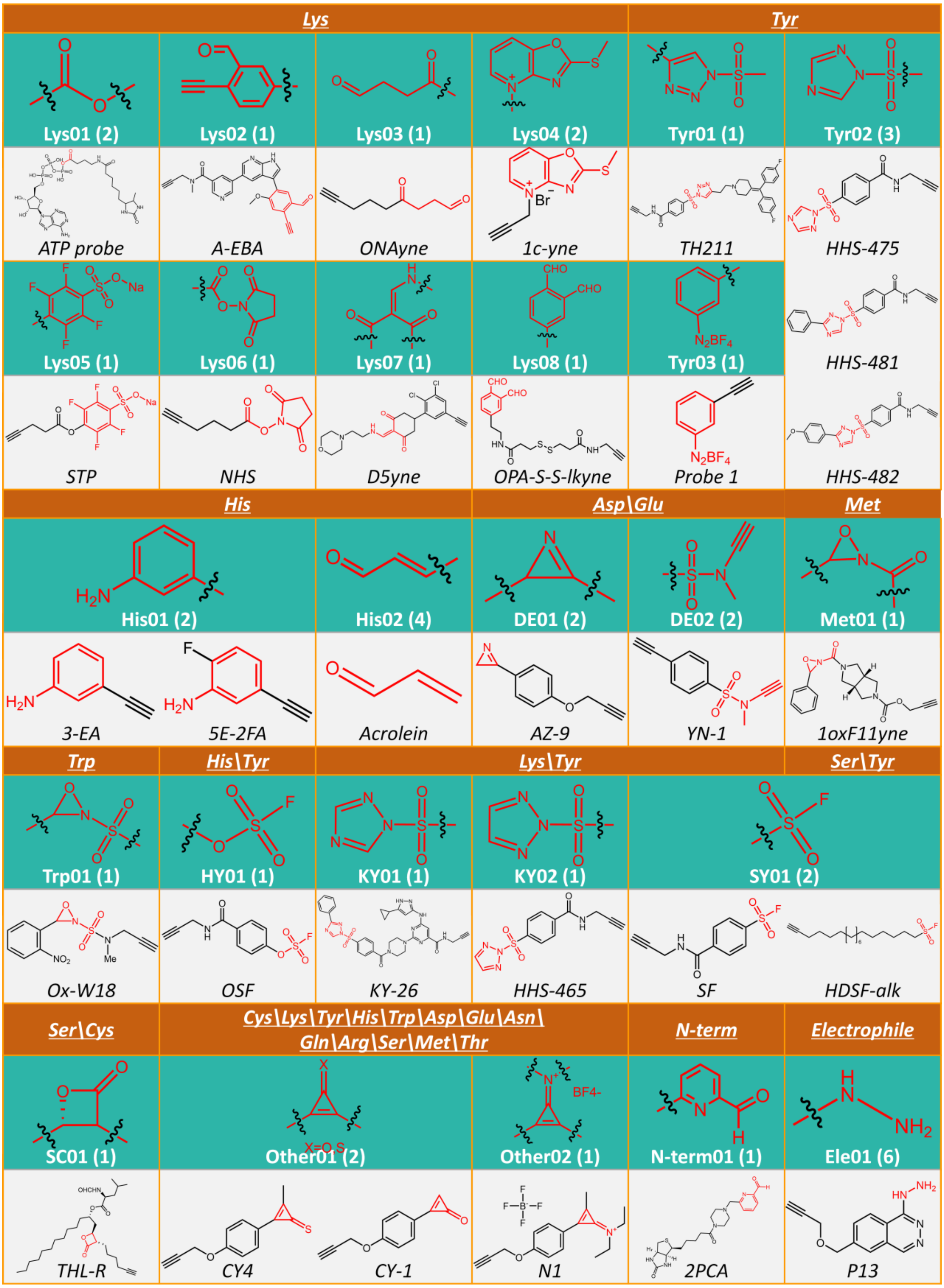

Figure S2. Atlas of *activity-based protein profiling* (ABPP) probe labelling the resides other than cysteine. There were a total of 26 types of ABPP probes (highlighted in *GREEN* background, the number in bracket indicated the total amount of ABPP probes within a specific probe type) from

13 classes (highlighted in *BROWN* background and *Italic* & *Underline* font). Particularly, **lysine** was an attractive residue for covalent ligand developments due to its intrinsically nucleophilic *ε*-amine group, its presence at numerous functional sites, and its frequent involvement in regulating protein structure and function through post-translational modifications. There were many broad-spectrum ABPP probes that labeled the residue lysine (such as gamma-dicarbonyl probe *ONAyne* and 2-methylthio pyridinium oxazoline ion probe *1c-yne*) and various tailor-made ABPP probes (such as kinase-targeting probes *ATP probe*). For **tyrosine**, specific probes were developed using sulfur-triazole exchange chemistry (such as *TH211*, *HHS-475* and *HHS-482*). As one of the most common catalytic residues, **histidine** was recently selectively labelled based on two-component strategy (such as *acrolein* and *3-EA*), while **carboxylic acid** residues (glutamic acid/aspartic acid) that were key for maintaining protein functions/structures, were selectively labelled using broad-spectrum probe (such as 2H-Azirine probe *AZ-9* and ynamide probe *YN-1*). As the key player in signaling pathways due to its oxidation state, **methionine** was selectively labelled by oxaziridine probe *1oxF11yne* in live cells, and **tryptophan**, with its unique roles in biology, were selectively labelled by N-sulfonyl oxaziridine probe *Ox-W18* in living systems. Moreover, other amino acids (such as **serine**, **threonine**, **glutamine**, **asparagine**, and **arginine**) had also been labelled by less selective probes, and the probes for proteolytic N-termini and protein electrophile cofactors were also included. For each of the 26 types (highlighted in *GREEN* background), both structures and names of the representative probes were explicitly offered, and the corresponding probe warhead was highlighted using *RED* color. A square frame in *ORANGE* was used to highlight the specific information for each ABPP probe type.

Table S1 Key differences between probes in Chem(Pro)^2 and the Chemical Probes Portal

	Chem(Pro)^2	Chemical Probes Portal
Applications of probes	Labeling and enriching target proteins for proteomic analysis, to discover both binding proteins and modes of action for studied compounds	Serving as inhibitor, activators or degraders, to ask mechanistic and phenotypic questions about a target in cell-based or animal studies.
Property requirement of probes toward target proteins	Both broad-spectrum and specific chemoproteomic probes are invaluable	Selectivity and potency, are essential attributes of chemical probes.
